# New mimarachnids (Hemiptera, Fulgoromorpha, Fulgoroidea) in mid-Cretaceous Burmese amber

**DOI:** 10.3897/zookeys.1057.66434

**Published:** 2021-08-25

**Authors:** Xiao Zhang, Yunzhi YaoDong Ren2, Hong Pang1, Huayan Chen1

**Affiliations:** 1 State Key Laboratory of Biocontrol, School of Life Sciences/School of Ecology, Sun Yat-sen University, Guangzhou 510275, China Sun Yat-sen University Guangzhou China; 2 College of Life Sciences and Academy for Multidisciplinary Studies, Capital Normal University, Xisanhuanbeilu 105, Haidian District, Beijing 100048, China Capital Normal University Beijing China

**Keywords:** fossil, palaeodiversity, planthopper, taxonomy, wing pigmentation

## Abstract

A new genus and species, *Multistriaorthotropa***gen. et sp. nov.**, and a new species, *Dachibangushui***sp. nov.**, of Mimarachnidae are described from the mid-Cretaceous Burmese amber. These new taxa display unique wing color patterns and extend the Mesozoic diversity of Mimarachnidae. The evolution of wing venation, phylogenetic placement of Mimarachnidae, and anti-predation defenses of this family in Burmese amber forest are briefly discussed.

## Introduction

Mimarachnidae Shcherbakov, 2007 is a small, extinct family belonging to the diverse phytophagous superfamily Fulgoroidea. They are medium-sized to large planthoppers and are characterized by the following characters: mesonotum with double median carinae, remnants of sensory pits in the adults, tegmina and hind wings with simplified venation and irregular network of veinlets, and basal cell absent or weak (Brysz and Szwedo 2019). Historically, species of this family were considered as members of the “cixiidae-like” planthoppers (Bourgoin and Szwedo 2008), and some mimarachnids are specialized insects with peculiar and striking forms (Shcherbakov 2007; Jiang et al. 2018, 2019; Zhang et al. 2018).

Fossil Mimarachnidae currently consist of 17 described species in 12 genera distributed from high latitude regions to tropical equatorial regions in the Cretaceous of Eurasia (Bourgoin 2021). Two monotypic genera, *Mimarachne* Shcherbakov, 2007 and *Saltissus* Shcherbakov, 2007 were first described from the Lower Cretaceous Baissa of Russia (145–125 Ma), then *Nipponoridium* (Fujiyama 1978; Szwedo 2008) were described from the Lower Cretaceous Kuwajima of Japan (140–120 Ma) (Szwedo 2008); and two genera, *Mimamontsecia* Szwedo & Ansorge, 2015 and *Chalicoridulum* Szwedo & Ansorge, 2015 were found from the Lower Cretaceous north-eastern Spain (130.0–125.5 Ma). Most other fossil Mimarachnidae were discovered from the mid-Cretaceous Burmese Kachin amber (98.79 ± 0.62 Ma), including the genera of *Burmissus* Shcherbakov, 2017, *Dachibangus* Jiang, Szwedo & Wang, 2018, *Jaculistilus* Zhang, Ren & Yao, 2018, *Mimaplax* Jiang, Szwedo & Wang, 2019, *Ayaimatum* Jiang & Szwedo, 2020, *Cretodorus* Fu & Huang, 2020, and *Mimaeurypterus* Fu & Huang, 2021. In addition, some specimens from the Cretaceous of Siberia, Mongolia, and Kazakhstan were mentioned by Shcherbakov (2007) but not formally described.

Herein, we describe a new genus with a new species, *Multistriaorthotropa* gen. et sp. nov., and a new species, *Dachibangushui* sp. nov., of Mimarachnidae from the mid-Cretaceous Myanmar. Both new species possess well-preserved wing venation and color pattern.

## Materials and methods

The specimens (contributed by Mr Zhengkun Hu) described in this study are from the Burmese amber collected from Hukawng Valley of Kachin in northern Myanmar (Li et al. 2017). The age of the Burmese amber is estimated to be the earliest Cenomanian, ~98.79 ± 0.62 Ma (Shi et al. 2012). Burmese amber from this site contains diverse insects (Ross 2021). The type specimens are housed in the Key Lab of Insect Evolution and Environmental Changes, College of Life Sciences, Capital Normal University, Beijing, China (CNUB; Yunzhi Yao, Curator).

The amber specimens were examined and photographed using a Nikon SMZ 25 microscope with an attached Nikon DS-Ri 2 digital camera system. The line drawings were made with Adobe Illustrator 2020 and Adobe Photoshop 2020. The wing venational nomenclature follows that of Bourgoin et al. (2015).

## Systematic palaeontology

### Order Hemiptera Linnaeus, 1758

**Suborder Fulgoromorpha Evans, 1946**

**Superfamily Fulgoroidea Latreille, 1807**

**Family Mimarachnidae Shcherbakov, 2007**

#### Genus Multistria

Taxon classificationAnimaliaHemipteraMimarachnidae

Zhang, Yao & Pang gen. nov.

66B80DE9-05E7-55C1-9CCE-C6A0DBE6170E

http://zoobank.org/50100984-8987-45c5-8429-de636befb635

Figures 1 , 2

##### Etymology.

The generic name is a combination of Latin “*multi*-” meaning “many” and “*stria*” meaning “streak”, referring to its wrinkled posterior pronotum. Gender feminine.

##### Type species.

*Multistriaorthotropa* Zhang, Yao & Pang, sp. nov.

##### Diagnosis.

Pronotum with posterior area rugulose (not rugulose in *Dachibangus*). Tegmen costal area narrow, exceeding 2/3 length of the wing, ScP + RA and RP single, close to each other, subparallel, MP with three terminals (no fewer than five terminals in *Dachibangus*), CuA forked early, near wing basal 1/3, CuA_2_ slightly curved mediad at level of tornus (more curved in *Dachibangus*). Without narrow marginal membrane. Hind wing CuA with two terminals.

**Figure 1.**
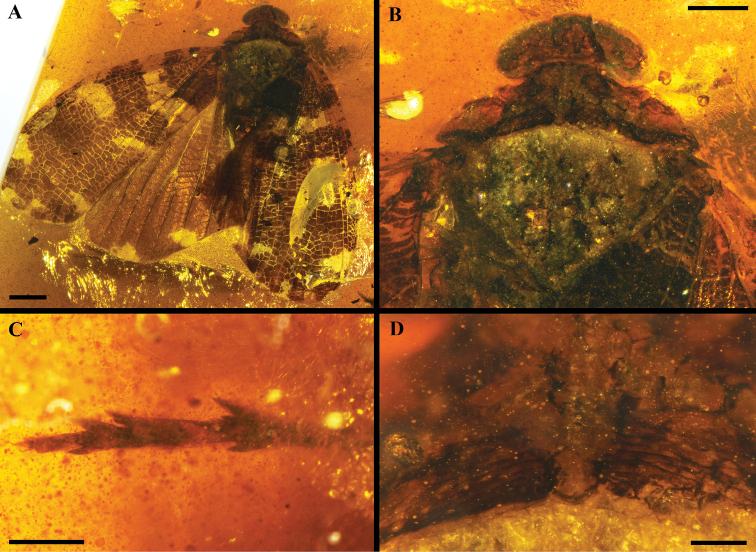
Holotype of *Multistriaorthotropa* gen. et sp. nov. **A** habitus in dorsal view **B** head and thorax in dorsal view **C** hind tarsus **D** pronotum in dorsal view. Scale bars: 2 mm (**A**); 1 mm (**B, C**); 0.25 mm (**D**).

**Figure 2.**
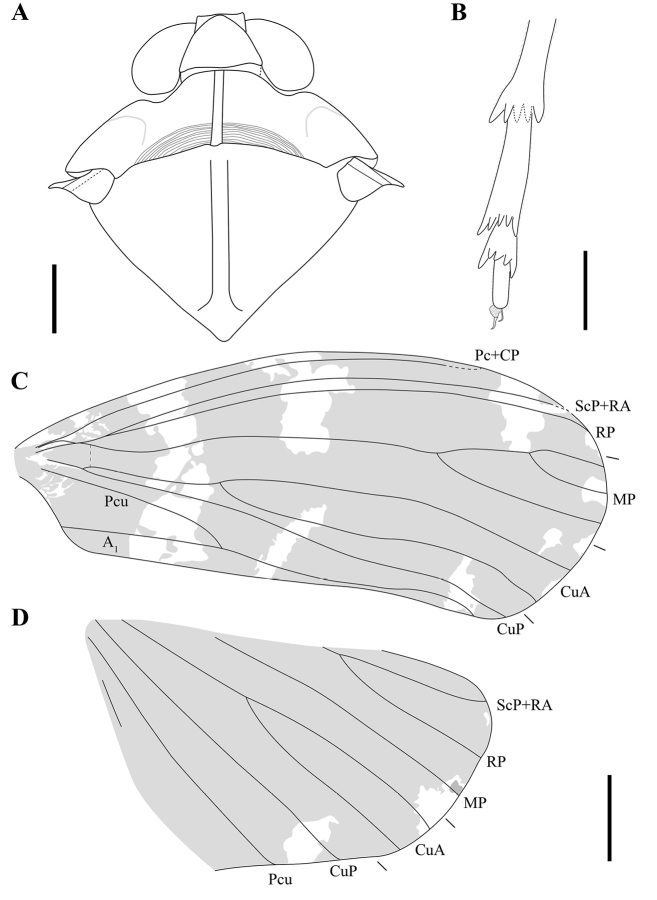
Line drawings of *Multistriaorthotropa* gen. et sp. nov. **A** head and thorax **B** hind tarsus **C** forewing **D** hind wing. Scale bars: 1 mm (**A, B**); 2 mm (**C, D**).

#### Multistria orthotropa

Taxon classificationAnimaliaHemipteraMimarachnidae

Zhang, Yao & Pang sp. nov.

4FDFBECD-315D-5EB9-B33B-962168B8D968

http://zoobank.org/335c6c62-d3f0-440e-8cce-036e63ac8f42

Figures 1 , 2

##### Etymology.

The specific name is from a Latin word “*orthotropus*” meaning “straight”, referring to its median carinae of mesonotum straight.

##### Type material.

***Holotype*,**CNU-HOM-MA2021001, gender unknown, a complete specimen but ventral view not visible.

##### Locality and horizon.

Hukawng Valley, Kachin State, Myanmar; mid-Cretaceous, lowermost Cenomanian.

##### Diagnosis.

Pronotum with anterior margin almost straight, posterior margin slightly concave, median carinae of mesonotum straight. Tegmen without spots, common stem ScP + R shorter than basal cell, Pcu almost straight, free part of Pcu distinctly shorter than common stem of Pcu + A_1_. Metatibio-metatarsal formula 5: 5: 5.

##### Description.

A well-preserved specimen, but ventral view not visible; total length of the holotype about 15.98 mm.

**Head**: head with compound eyes about 2.52 mm wide, wider than half of pronotum width. Vertex triangular, without median carina, lateral margins carinate, posterior margin sinuous, trigons visible in dorsal view.

**Thorax**: pronotum subhexagonal, length distinctly shorter than mesonotum, about 4.3 times as wide as long in midline, posterior area of pronotum rugulose, anterior margin almost straight, posterior margin arcuate and concave, median carinae double and parallel, present throughout, lateral carinae invisible. Mesonotum poorly preserved, wider than long in midline, median carinae parallel and paired, diverging laterad on scutellum, lateral carinae invisible, scutellum indistinct. Tegula subquadrate, large and distinctly carinate.

**Leg**: only part of hind leg visible, covered with short setae. Hind tibia widened apically, with five apical teeth; hind tarsi with three segments, basitarsomere 1.72 mm long, distinctly longer than combined length of midtarsomere and apical tarsomere, with five apical teeth, the external teeth longer than inner group; midtarsomere 0.89 mm long, with five apical teeth, the external teeth longer than inner group; subapical setae on all pectens invisible; apical tarsomere 0.67 mm long; tarsal claws developed, arolium wide.

**Wings**: membranous. Tegmen 14.03 mm long, 5.55 mm wide, about 2.5 times as long as wide, with distinct venation and irregular network veinlets, and also with irregular colour bands from base to apex, costal margin weakly arched at base, anteroapical and posteroapical angles broadly rounded, posterior margin straight, tornus present. Costal area narrow and long, with transverse veinlets, narrowing toward wing apex, basal cell weak, arculus indistinct, Pc + CP extends nearly to wing apex, apical portion invisible, common stem ScP + R + M longer than common stem ScP + R, branch ScP + RA and RP subparallel to costal margin, not forked, stem MP curved at base then almost straight, forked in wing apical half, with three terminals, branch MP_1+2_ forked, reaching margin with two terminals, branch MP_3+4_ simple, CuA forked near wing basal one-third, with two terminals, CuA_1_ basally subparallel to CuA_2_, CuA_2_ slightly curved mediad at level of tornus, CuP present throughout wing, slightly sinuate, clavus open, Pcu and A_1_ fused nearly at the same level of CuA forking, free part of Pcu distinctly shorter than common stem of Pcu + A_1_, narrow marginal membrane absent.

Hind wing membranous, about 11.01 mm long, 6.60 mm wide, slightly shorter than tegmen, coloration of hind wing darker, two lighter irregular spots near posteroapical portion, irregular network veinlets present. Anteroapical angle round, ScP + R forked, with two terminals, ScP + RA curved in apical portion, stem MP single, CuA forked at wing midlength, reaching margin with two terminals, CuP almost straight, Pcu slightly sinuous.

##### Remarks.

The new genus is assigned to Mimarachnidae based on the following characters: mesonotum with double median carinae, remnants of sensory pits at the adults, wings with simplified venation, and irregular network of veinlets, basal cell weak, hind wing MP simple. This new genus is distinguished from other genera by the following characters: posterior area of pronotum rugulose (vs no such character in the other known genera); tegmen costal area exceeding 2/3 length of the wing (vs less than ½ of wing length in *Chalicoridulum*, *Ayaimatum*, and *Mimaeurypterus*, costal area absent in *Mimaplax*); ScP + RA and RP single (vs ScP + RA and RP forked in *Mimarachne* and *Saltissus*, RP forked in *Mimamontsecia*); tegmen ScP + RA and RP close to each other, subparallel (vs ScP + RA diverging from RP in *Mimarachne*, *Saltissus*, *Chalicoridulum*, *Mimamontsecia*); MP with three terminals (vs single in *Cretodorus*, two terminals in *Saltissus*, *Chalicoridulum*, *Mimamontsecia*, *Burmissus*, and *Ayaimatum*, no fewer than four terminals in *Jaculistilus* and *Dachibangus*); CuA forked early, near wing basal 1/3 (forked late, near wing midpoint in *Mimaplax*, *Chalicoridulum*, *Saltissus*, *Burmissus*, *Ayaimatum*, *Cretodorus*); tegmen without narrow marginal membrane (vs with narrow marginal membrane in *Mimarachne*, *Chalicoridulum*, *Mimamontsecia*, *Burmissus*, *Cretodorus*, *Mimaeurypterus*); hind wing CuA with two terminals (vs three terminals in *Nipponoridium*).

#### Genus Dachibangus

Taxon classificationAnimaliaHemipteraMimarachnidae

Jiang, Szwedo & Wang, 2018

0F62EE59-7734-5AE3-9385-416646A33063

##### Type species.

*trimaculatus* Jiang, Szwedo & Wang, 2018; by original designation and monotype.

#### Dachibangus hui

Taxon classificationAnimaliaHemipteraMimarachnidae

Zhang, Yao & Pang sp. nov.

15A7F261-7E29-5A28-AF20-87999D865287

http://zoobank.org/24d479b0-ebb5-4c2f-95d3-3c1a41ff403a

Figures 3 , 4

##### Etymology.

The new specific name is dedicated to Mr Zhengkun Hu for his donation of the Burmese amber containing the holotype.

##### Type material.

***Holotype*,**CNU-HOM-MA2021002, adult male, wings well preserved, but legs missing.

##### Locality and horizon.

Hukawng Valley, Kachin State, Myanmar; mid-Cretaceous, lowermost Cenomanian.

##### Diagnosis.

Median carinae of mesonotum straight, subparallel to each other, lateral carinae posterior portion nearly straight (median carinae slightly sinuate, lateral carinae posterior portion arcuate in *D.trimaculatus*); tegmen without spots (with spots in *D.trimaculatus* and *D.formosus*); common stem ScP + R as long as basal cell (ScP + R longer than basal cell in *D.formosus*, ScP + R about 1/2 of basal cell in *D.trimaculatus*); MP with five terminals (six terminals in *D.trimaculatus*); the bifurcation of MP_1+2_ slightly proximad of the bifurcation of MP_3+4_ (the bifurcation of MP_1+2_ slightly apicad of the bifurcation of MP_3+4_ in *D.formosus*); CuA_1_ almost straight (arcuate in *D.trimaculatus*); CuA_2_ slightly curved mediad at level of tornus (more curved in *D.formosus*, strongly curved in *D.trimaculatus*); CuP almost straight (sinuate in *D.trimaculatus* and *D.formosus*); free stem of Pcu nearly as long as common stem of Pcu + A_1_ (Pcu longer than Pcu + A_1_ in *D.trimaculatus*); CuP and Pcu + A_1_ not close to each other (close to each other in *D.trimaculatus*); hind wing CuA forked at wing midlength (forked near wing base in *D.trimaculatus*).

**Figure 3.**
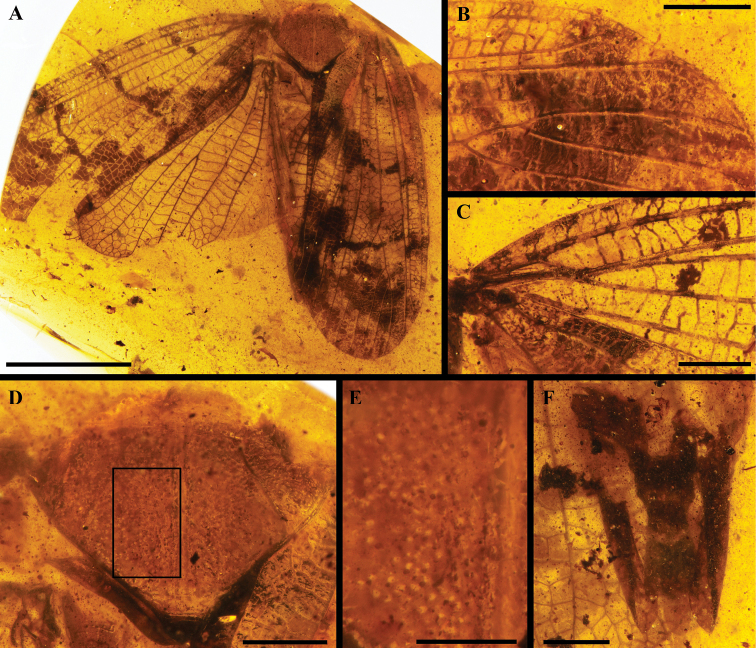
Holotype of *Dachibangushui* sp. nov. **A** habitus in dorsal view **B** distal part of forewing **C** basal part of forewing **D** mesonotum **E** sensory pits on mesonotum **F** male terminalia in ventral view. Scale bars: 5 mm (**A**); 1 mm (**B, C, D, F**); 0.5 mm (**E**).

**Figure 4.**
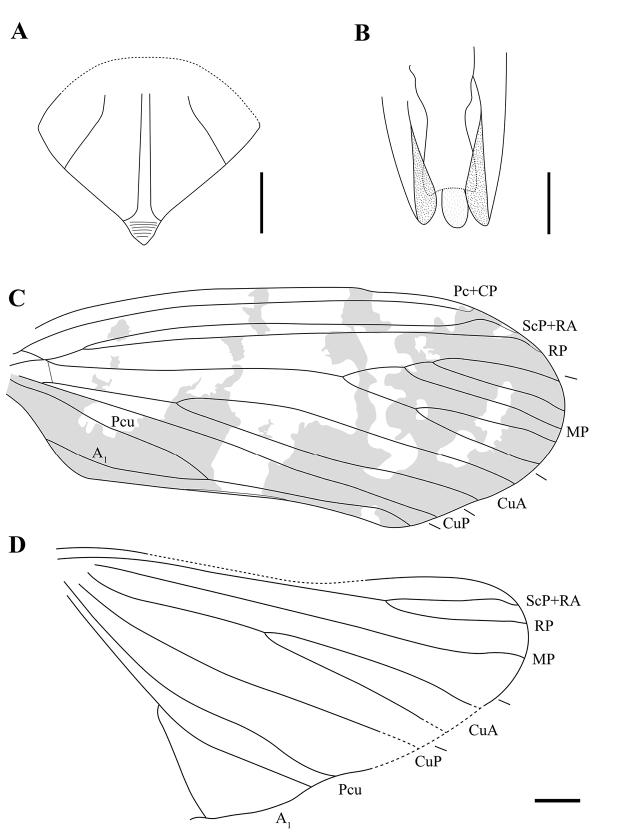
Line drawings of *Dachibangushui* sp. nov. **A** mesonotum **B** male terminalia **C** forewing **D** hind wing. Scale bars: 1 mm (**A–D**).

##### Description.

Total length of the preserved holotype about 14.21 mm, wings well-preserved.

**Thorax**: mesonotum wider than long in midline, densely punctate, median carinae paired, diverging laterad on scutellum, lateral carinae distinct, not reaching anterior margin, diverging posteriad, scutellum transversely wrinkled. Tegula large.

**Wings**: membranous. Tegmen 13.59 mm long, 5.06 mm wide, about 2.7 times as long as wide, with distinct venation and irregular network veinlets, and also with irregular colour bands from base to apex, costal margin weakly arched at base, apical margin round, posterior margin almost straight, tornus distinct. Costal area narrow and long, with transverse veinlets, narrowing toward tegmen apex, basal cell weak, arculus weak, Pc + CP parallel to costal margin, apical portion weakened, common stem ScP + R + M approximately as long as common stem ScP + R, ScP + RA not forked, posterior portion curved upward, then downward to apical margin, apical portion weakened, RP single, apical portion weakened, stem MP almost straight, forked in wing apical half, with five terminals, the bifurcation of MP_1+2_ slightly proximad of the bifurcation of MP_3+4_, MP_1+2_ reaching margin with three terminals, MP_3+4_ with two terminals, CuA forked near wing basal one-third, with two terminals, CuP present throughout wing, nearly straight, clavus open, Pcu and A_1_ fused apicad of CuA forking, narrow marginal membrane absent, wing-coupling fore fold present.

Hind wing membranous, about 10.57 mm long, 5.63 mm wide, slightly shorter than tegmen, without distinct coloration, irregular network veinlets present. Anteroapical angle round, stem ScP + R straight, forked late, with two terminals, ScP + RA apical portion curved, MP single, CuA forked at wing midlength, with two terminals, CuP almost straight, apical portion absent, Pcu weakly sinuous, A_1_ forked, giving off two branches.

**Abdomen**: male terminalia poorly preserved, with two symmetrical lobes, pygofer lobes carinate, anal tube elongate, anal styles protruding and ligulate.

##### Remarks.

The new species is attributed to the genus *Dachibangus* due to mesonotum median carinae diverging laterad on scutellum, lateral carinae strongly diverging posteriad, tegmen with irregular colour bands, costal area narrow, ScP + RA curved downward in apical portion, MP at least with five terminals, CuA_2_ curved mediad at level of tornus, tornus distinct.

## Discussion

Including the new taxa described in this study, Mimarachnidae now comprise 13 genera and 19 species, all confined to the Cretaceous. Among them, five genera and five species have been described from the early Cretaceous of Russia, Spain, and Japan, and the rest were discovered from the mid-Cretaceous Burmese amber. During early Cretaceous period, tegmen ScP + RA and RP of mimarachnids were generally forked, with the MP having 2 or 3 terminals, such as in *Mimarachne*, *Saltissus*, and *Mimamontsecia*. However, by the mid-Cretaceous, tegmen ScP + RA, and RP were unbranched (all species) and the MP single (*Cretodorus*) or with 2 or 3 terminals (*Burmissus*, *Mimaplax*, *Ayaimatum*, and *Mimaeurypterus*) or with no fewer than 4 terminals (*Jaculistilus* and *Dachibangus*). Therefore, we speculate that the number of tegmen ScP + R terminals gradually reduced, and the number of MP terminals seems to have been diversified during the evolutionary process of Mimarachnidae.

The placement of Mimarachnidae in Fulgoroidea remains unclear. Shcherbakov (2007) placed Mimarachnidae in the “pre-cixioid section of Fulgoroidea” and related them to Perforissidae. Subsequently, this family was generally assigned into the “cixiidae-like” planthopper group (Bourgoin and Szwedo 2008; Szwedo and Ansorge 2015; Brysz and Szwedo 2019), which is an informal group comprises some extinct and extant families similar to Cixiidae. Mimarachnidae are unique planthoppers in the Mesozoic. Mimarachnidae and Perforissidae share many similar characters such as the simplified venation, remnants of sensory pits at the adults, destabilization of hind leg armature (Shcherbakov 2017). But, as Jiang et al. (2018) suggested, these similarities are also shared by various families of Fulgoroidea. These similarities cannot support a close relationship between Mimarachnidae and Perforissidae because they could also result from convergent or parallel evolution. Besides, Mimarachnidae and the “cixiidae-like” families show obvious differences in the morphology, such as venation patterns, shape of head and thorax, and number of carinae, which suggests that they do not form a lineage and share a common ancestor. A robust placement of Mimarachnidae in Fulgoroidea still needs further study.

Fulgoromorpha are phytophagous insects. These planthoppers stay on the host plants for a long time to suck fluids, with wings covering their bodies. Colour pattern of the wings might have become important for serving as a defensive strategy to disguise themselves from enemies. In *Multistria* gen. nov. and *Dachibangus*, the tegmina are covered with irregular color bands from the base to the apex, contrasting highly and extending to the tegmina edges. This disruptive coloration could effectively break up the shape and destroy the outline of the insects (Stevens and Merilaita 2009a, 2009b), and thereby make these larger planthoppers more difficult to be detected. Similarly, in *D.trimaculatus* Jiang, Szwedo & Wang, 2018, *D.formosus* Fu, Szwedo, Azar & Huang, 2019, and *Jaculistilusoligotrichus* Zhang, Ren & Yao, 2018, the dark wing spots are obvious and may also have anti-predation function (Théry and Gomez 2010). In addition, *Mimaplaxekrypsan* Jiang, Szwedo & Wang, 2019 used more complicate camouflaged configuration to avoid possible predation. It is possible that mimarachnids have evolved with a range of anti-predation defenses in Burmese amber forests, such as disruptive coloration, wing spots, and “flatoidinisation syndrome” (a specialized and complex camouflage, uniting shape, colour, and behaviour) (Jiang et al. 2019) to help them to avoid being attacked by predators.

## Supplementary Material

XML Treatment for Multistria

XML Treatment for Multistria orthotropa

XML Treatment for Dachibangus

XML Treatment for Dachibangus hui
